# Functional Outcomes with Facial Artery Musculo-Mucosal (FAMM) Flap and Dental Implants for Reconstruction of Floor of the Mouth and Tongue Defects in Oncologic Patients

**DOI:** 10.3390/jcm10163625

**Published:** 2021-08-17

**Authors:** Carlos Navarro Cuéllar, Manuel Tousidonis Rial, Raúl Antúnez-Conde, Marc Agea Martínez, Ignacio Navarro Cuéllar, José Ignacio Salmerón Escobar, Carlos Navarro Vila

**Affiliations:** Maxillofacial Surgery Department, Hospital General Universitario Gregorio Marañón, C/Doctor Esquerdo 46, 28007 Madrid, Spain; manuel@tousidonisrial.com (M.T.R.); antunezconde_92@hotmail.com (R.A.-C.); ageamarc@gmail.com (M.A.M.); nnavcu@hotmail.com (I.N.C.); jisalmeron@telefonica.net (J.I.S.E.); hazamachado2@gmail.com (C.N.V.)

**Keywords:** FAMM flap, dental implants, floor of the mouth and tongue defects, oncologic patients

## Abstract

Optimal functional outcomes in oncologic patients with squamous cell carcinoma (SCCA) of the tongue and floor of the mouth require good lingual mobility, adequate facial competence, the cheek suction effect and dental rehabilitation with osseointegrated implants. In this study, twenty-two oncologic patients who had been diagnosed with intraoral SCCA affecting the tongue and the floor of the mouth and who had undergone wide resection of the tumor and immediate reconstruction with an inferiorly pedicled FAMM flap and immediate osseointegrated implants were assessed. Lingual mobility, speech articulation, deglutition, implant success rate, mouth opening, and aesthetic results were evaluated. All patients were staged as T2 and the defect size ranged from 3.7 × 2.1 cm to 6.3 × 4.2 cm. A selective neck dissection was performed in all patients as part of their oncologic treatment, either electively or for node positive disease. Thirteen patients (59%) were diagnosed with node positive disease and underwent adjuvant radiotherapy. A total of 101 osseointegrated implants were placed for prosthetic rehabilitation and 8 implants were lost (7.9%), of which 7 received radiotherapy (87.5%). The implant success rate was 92.1%. Mouth opening was reported as normal in 19 patients (86.3%). Tongue tip elevation was reported as excellent in 19 patients (86.3%) and good in 3 patients (13.6%). Lingual protrusion was referred to as excellent in 15 patients (68.2%) and good in 6 patients (27.2%). Lateral excursion was reported as excellent in 14 patients (63.6%) and good in 7 patients (31.8%). In terms of speech articulation, 20 patients reported normal speech (90.9%). Regarding deglutition, 19 patients (86.3%) reported a regular diet while a soft diet was reported by 3 patients (13.7%). Aesthetic results were referred to as excellent in 17 patients (77.3%). FAMM flaps, immediate implants and fixed prostheses enable the functional rehabilitation of oncologic patients, optimizing aesthetics and functional outcomes even in patients undergoing irradiation, thus returning oncologic patients to an excellent quality of life.

## 1. Introduction

The surgical management of squamous cell carcinoma (SCCA) involving the tongue and the floor of the mouth (FOM) may lead to complex defects that require immediate reconstruction to reestablish form and function [1]. The ablative and reconstructive surgeries can be particularly challenging and can be approached in several ways depending on the extension of the tumor, the nodal staging, and the involvement of other structures. Wide resection of intraoral SCCA with clear margins often leads to poor anterolateral mobility of the tongue and can impact speech and deglutition [2]. Small intraoral defects can be closed primarily or heal by secondary intention. For large defects, free flaps remain the gold standard due to the amount of tissue they provide, and their reliability and high rate of success [3]. Nevertheless, medium-sized defects of the floor of the mouth and up to one third of the tongue can be accurately reconstructed with pedicled flaps with limited morbidity to the donor site. The facial artery musculomucosal (FAMM) flap was first described by Pribaz [4] as being suitable for the reconstruction of a wide range of intraoral surgical defects. The FAMM flap provides mucosa, submucosa, and buccinator muscle and can be superiorly pedicled on the angular artery with a retrograde flow or inferiorly pedicled with an anterograde flow on the facial artery. It is a safe, thin and reliable flap with a great axis of rotation for intraoral reconstruction even in previous radiated patients [5]. On the other hand, molar extraction is required to avoid the biting of the pedicle and a second-stage procedure may be required to section the pedicle.

The restoration of function after oncologic surgery of the tongue and FOM is a challenge still facing head and neck oncology. In oncologic surgery of the oral cavity, conventional dental rehabilitation offers a low success rate because of the existing distortion of the intraoral anatomy and the adverse effects of radiotherapy [6]. Osseointegrated implants have afforded major advances in the reconstructive management of oncologic patients because they allow stabilization of dental prostheses. In this way patients can be offered optimal reconstruction by securing true aesthetic and functional rehabilitation.

The aim of this study was to evaluate the functional outcomes of oncologic patients diagnosed with intraoral SCCA who underwent resection of the tongue and floor of mouth and immediate reconstruction with a FAMM flap and osseointegrated implants. The specific aims of this study were: (1) to evaluate lingual mobility; (2) to evaluate speech articulation; (3) to evaluate deglutition; (4) to evaluate the implant success rate; (5) to evaluate mouth opening; (6) to evaluate aesthetic results.

## 2. Materials and Methods

To address the research purpose, the investigators designed and implemented a retrospective study during a 7-year period (2012–2018) with twenty-two oncologic patients (15 men and 7 women) diagnosed with intraoral SCCA affecting the tongue and the floor of the mouth. All patients underwent wide resection of the tumor and immediate reconstruction with an inferiorly pedicled FAMM flap and osseointegrated implants (Ticare, Valladolid, Spain). In edentulous patients, implants were placed both in the mandible and in the maxilla to achieve optimal functional reconstruction. In dentate patients who required extraction of the last molars, to avoid damaging the flap, dental implants were placed at the same time as tooth extraction. The patients were staged according to the TNM classification clinically and radiologically using CT or MRI. A selective neck dissection with preservation of the facial vessels was performed in all patients as part of their oncologic resection, either electively or for node positive disease. In non-irradiated patients, prosthetic rehabilitation was performed 4 months after reconstructive surgery. In irradiated patients, dental rehabilitation was deferred until 8 months after the end of radiotherapy. All the patients were treated by the Department of Maxillofacial Surgery at Gregorio Marañón Hospital in Madrid, Spain. The study and review of the medical records and data collection and the subsequent analysis of the data collected were endorsed by the Hospital Ethics Committee. The inclusion criteria were: (1) histologic diagnosis of primary SCCA affecting the floor of the mouth and/or tongue; (2) pathologically staged as T2; (3) patients reconstructed with inferiorly based FAMM flap; (4) edentulous patients with immediate implant placement; (5) dentate patients who underwent tooth extraction and immediate implants; (6) prosthetic rehabilitation with an implant-fixed prosthesis; (7) a minimum follow-up of two years. The exclusion criteria were: (1) defects greater than one third of the tongue for which reconstruction with a free flap was performed; (2) mandibular invasion requiring segmental mandibulectomy; (3) patients previously treated surgically or with radiotherapy in the affected area; (4) patients with distant metastases at the time of diagnosis.

The variables evaluated in this study were:

1. Implant success rate evaluated two years after prosthetic rehabilitation. The criteria for implant success were immobility, absence of peri-implant radiolucency, adequate width of the attached gingiva, and absence of infection.
2. Mouth opening: it was evaluated as normal (N), partly limited (PL) and limited (L). Normal mouth opening was considered ≥40 mm. Partly limited was classed as a 30–39 mm range, and limited was classed as a mouth opening ≤30 mm.
3. Lingual mobility: we used the tongue mobility scale published by Jowett [3], graded according to tip elevation with an open mouth (excellent—to hard palate, good—to upper dentition, poor—below upper dentition), tip protrusion (excellent—extends past lower vermillion border, good—extends to lower vermillion border, fair—extends to lower dentition, poor—fixed to the floor of the mouth) and maximal lateral movement of the tip (excellent—extends to oral commissure on both sides, good—approaches oral commissure on the other side, fair—approaches oral commissure on both sides, poor—the tip does not approach oral commissure on either side).
4. Speech articulation was evaluated as normal (N), partly intelligible (PI), or unintelligble (U). Speech articulation was evaluated subjectively by the patient and the maxillofacial surgeon by comparing the preoperative and postoperative situation. Unlike studies performed for speech evaluation in adults in different areas of oral surgery and orthognathic surgery [7,8], in this study no speech evaluation was performed by a speech pathologist.
5. Deglutition was assessed and results were classified with scores of 0 (liquid diet), 1 (soft diet) and 2 (regular diet).
6. Aesthetic results: aesthetic assessment by the patients was subjectively addressed by comparing the preoperative and postoperative situation regarding the symmetry of the commissure position at rest, and for the smile, comparing the affected side with the non-affected side, and the aesthetic results of the implant rehabilitation. The aesthetic results were assessed as excellent (E), good (G), acceptable (A) and poor (P).

### Surgical Technique

All FAMM flaps were inferiorly pedicled for tongue and floor of the mouth reconstruction. We did not use Doppler to identify the facial artery, but the flap was designed over the anatomic facial artery trajectory. The flap was elevated from the retromolar trigone to the ipsilateral labial sulcus. The anterior border of the flap was positioned 1 cm posterior to the oral commissure to avoid distortion of the commissure. The flap was limited posteriorly by Stensen’s duct. Inferiorly, the flap base was 2 to 3 cm large and centered over the area of the second and third molars. Flap elevation began 1 cm lateral to the oral commissure, and mucosa, submucosa and buccinator muscle were incised to identify the facial artery. The flap, along with the buccinator muscle and a small portion of the orbicularis ori, were elevated posteroinferiorly, keeping the facial artery attached to the overlying tissues in its entire length. The inclusion of the facial vein was not necessary as the venous drainage relies on the submucosal plexus. As far as possible, we used the modification of the surgical approach published by Duranceau and Ayad [9] in which the pedicle was used to fill the posterior part of the FOM defect by extending the anterior incision over the alveolar crest in order to reach the FOM defect. Through this approach, a second stage procedure was avoided. Nevertheless, in patients in which this modification was not performed, a second-stage procedure under local anesthesia was accomplished 3 weeks after the initial surgery. Prior to defect closure, the posterior molars were removed and osseointegrated implants were immediately placed. In edentulous patients or in patients with a severe periodontal disease, implants were placed in the remnant mandible and maxilla to achieve prosthetic rehabilitation. In non-irradiated patients, prosthetic rehabilitation was performed 4 months after reconstructive surgery. In irradiated patients, dental rehabilitation was deferred until 8 months after the end of radiotherapy.

## 3. Results

Twenty-two oncologic patients diagnosed with SCCA of the floor of the mouth and tongue were reconstructed with a FAMM flap and immediate osseointegrated implants. The follow-up period was from 2 years 5 months to 7 years 3 months (average 4 years 9 months). Fifteen patients were male (68.2%), and seven patients were female (31.8%), with a mean age of 62.4 years (range: 51 to 72 years). All patients were staged as T2 based on the TNM classification and underwent resection with free margins (Table 1). 

The defect size ranged from 3.7 × 2.1 cm to 6.3 × 4.2 cm. All FAMM flaps were inferiorly pedicled for tongue and floor of the mouth reconstruction. Fifteen patients (68.2%) required a second-stage procedure to take down and inset the flap pedicle 3 weeks after initial surgery under local anesthesia. A selective neck dissection (levels 1–3 or 1–4) was performed in all patients as part of their oncologic treatment, either electively or for node positive disease. Regarding pathological N staging, 13 patients (59%) were diagnosed with node positive disease. pT2N1 was diagnosed in 7 patients, while pT2N2a was diagnosed in 3 patients and PT2N2b in 3 patients. All patients with node positive disease underwent adjuvant radiotherapy (59%). Regarding complications, in 16 patients there were no complications (72.7%). We reported two donor site dehiscences, two patients with venous congestion and one hematoma, all of which were treated conservatively. One patient developed partial distal flap necrosis that required surgical debridement. A total of 101 osseointegrated implants (Ticare^®^, Valladolid, Spain) were placed, with an average of 4.59 implants per patient. A total of 8 implants were lost (7.9%), of which 7 received radiotherapy (87.5%). Nevertheless, the rest of the implants (93 implants) were loaded and rehabilitated with implant-fixed prostheses. The implant success rate was 92.1%. Mouth opening was reported as normal in 19 patients (86.3%) and partially limited in 3 patients (13.6%). Tongue tip elevation was reported as excellent in 19 patients (86.3%) and good in 3 patients (13.6%). Lingual protrusion was referred to as excellent in 15 patients (68.2%), good in 6 patients (27.2%) and fair in 1 patient. Lateral excursion was reported as excellent in 14 patients (63.6%), good in 7 patients (31.8%) and fair in 1 patient. In terms of speech articulation, 20 patients reported normal speech (90.9%) while 2 patients reported partly intelligible speech. Regarding deglutition, 19 patients (86.3%) reported a regular diet while a soft diet was reported in 3 patients (13.7%). Aesthetic results were referred to as excellent in 17 patients (77.3%), good in 4 patients and acceptable in 1 patient. 

### Case Presentation

**Figure 1:**
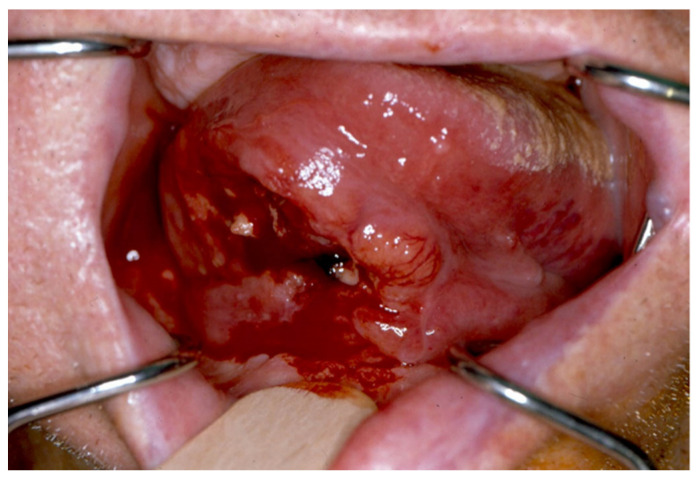
Squamous cell carcinoma (SCCA) involving the right tongue and the floor of the mouth.

A 59-year-old male patient, a severe smoker, was diagnosed with squamous cell carcinoma of the right tongue with ipsilateral floor of the mouth involvement (T2N2bM0) (Figure 1). 

**Figure 2:**
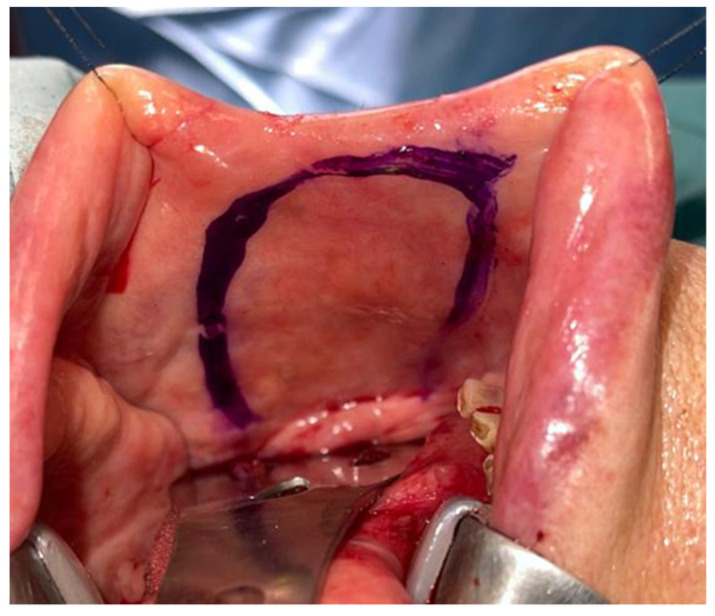
Design of the inferiorly pedicled FAMM flap.

A homolateral selective cervical dissection with resection of the tumor of the tongue and floor of the mouth with free margins was performed. The defect to be reconstructed was 5.7 × 3.6 cm and a FAMM flap was designed for reconstruction (Figure 2). 

**Figure 3:**
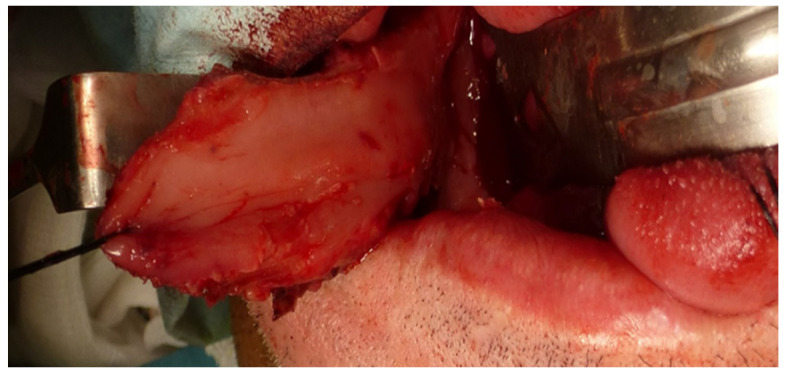
Right FAMM flap inferiorly pedicled with an anterograde flow on the facial artery.

**Figure 4:**
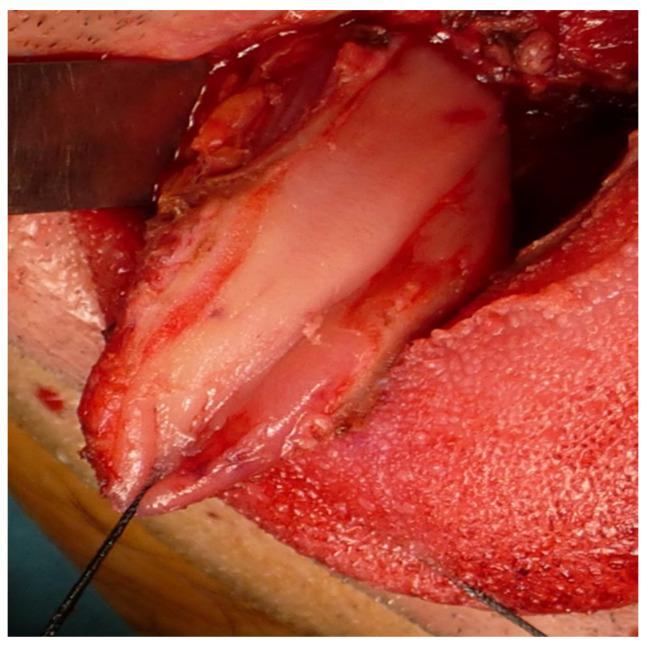
Immediate reconstruction of tongue and floor of the mouth defect.

**Figure 5:**
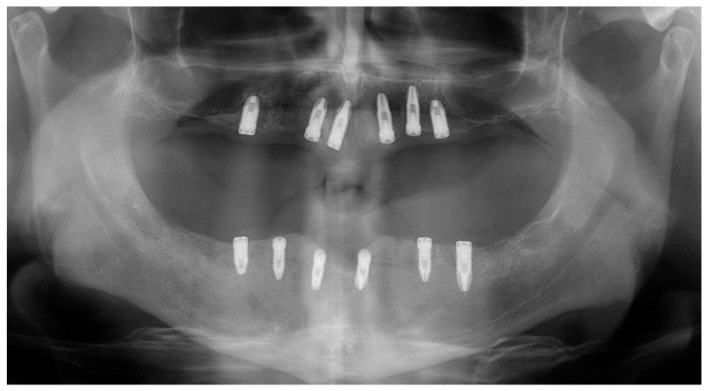
Immediate dental implants in both the maxilla and mandible for functional rehabilitation after teeth extraction.

Immediate reconstruction of the defect was performed with an inferiorly based FAMM flap (Figure 3 and Figure 4) and immediate placement of 12 dental implants, 6 in the mandible and 6 in the maxilla (Figure 5). There were no postoperative complications, and the patient received postoperative radiotherapy (60 Gy). Eight months later, prosthetic rehabilitation of the dental implants was performed, and the aesthetic and functional evaluations were accomplished. 

**Figure 6:**
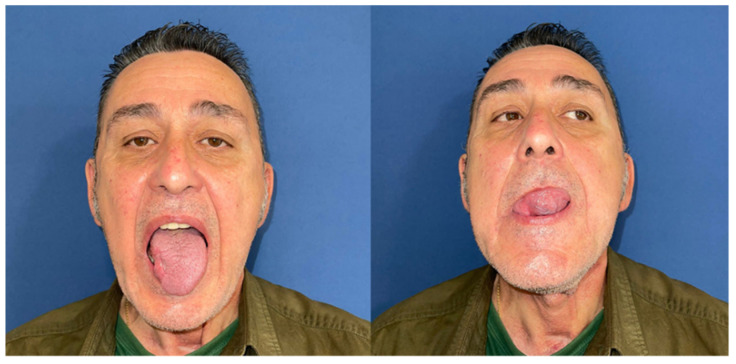
Aesthetic and functional results eight months after surgery with a FAMM flap for right tongue and floor of the mouth defect. Functional outcomes with lingual mobility: excellent tip elevation with open mouth and excellent tip protrusion, extending past lower vermillion border.

**Figure 7:**
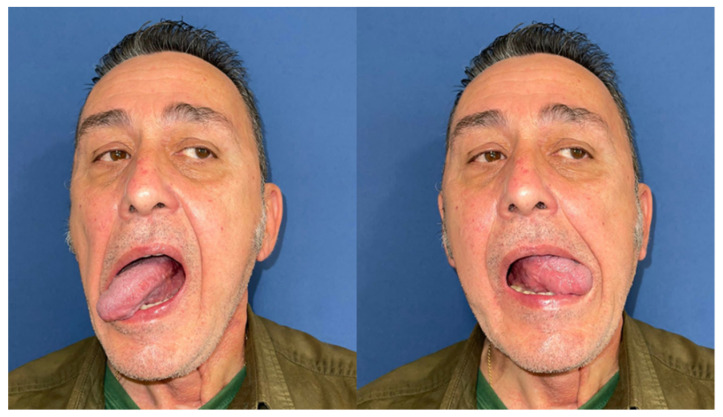
Excellent maximal lateral movement of the tip extending to the oral commissure on both sides.

**Figure 8:**
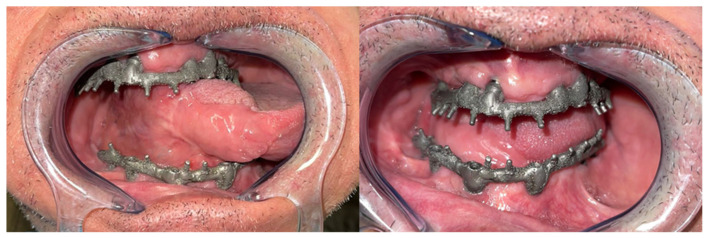
Metal framework evaluated intraorally for functional rehabilitation with a fixed implant-supported prosthesis. FAMM flap for right tongue and floor of the mouth reconstruction.

**Figure 9:**
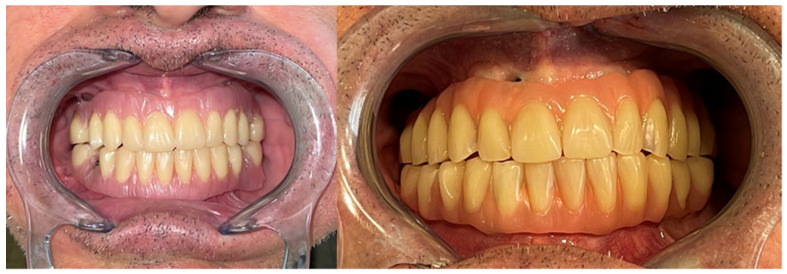
Prosthetic rehabilitation with a fixed implant-supported prosthesis and final occlusion.

**Figure 10:**
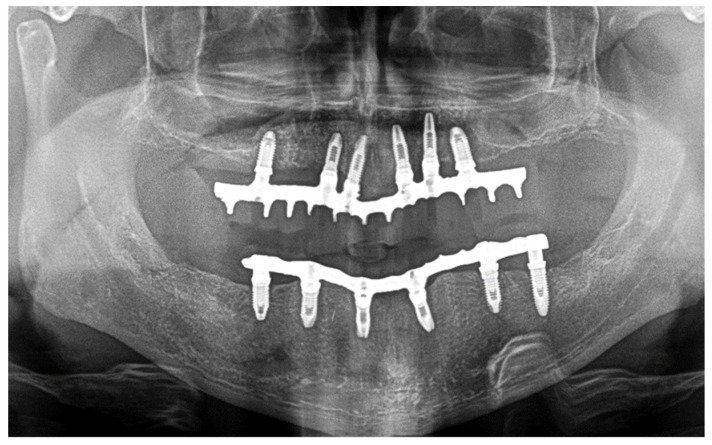
Panoramic radiograph after prosthetic rehabilitation with an implant-fixed prosthesis.

**Figure 11:**
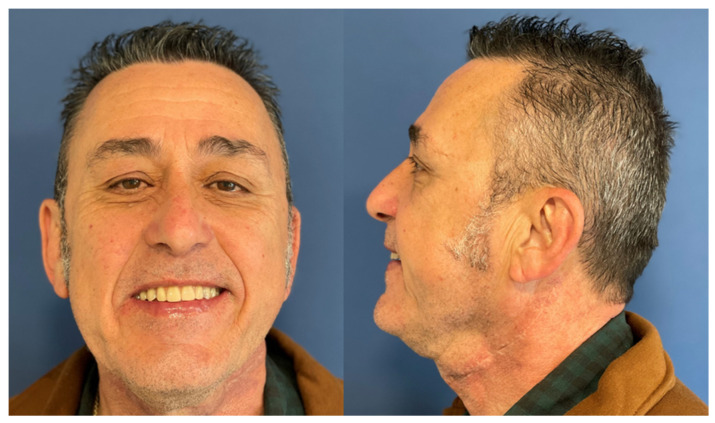
Aesthetic results eight months after surgery with a FAMM flap and immediate dental implants for tongue and floor of the mouth defect.

Mouth opening was evaluated as normal and lingual mobility as excellent for tip elevation, lingual protrusion, and lateral movements (Figure 6 and Figure 7). The patient was rehabilitated with two implant-supported prostheses (Figure 8, Figure 9 and Figure 10). Their speech articulation was evaluated as normal and the patient reported a normal diet without restrictions. From the aesthetic point of view, the patient reported an excellent result (Figure 11).

## 4. Discussion

The main goal when approaching SCCA of the floor of the mouth and tongue are oncological outcomes in terms of free resection margins and patient survival [10]. Nevertheless, the restoration of function after oncologic surgery of the oral cavity remains one of the major challenges facing head and neck oncology [6]. The anatomic alterations resulting from tumor resection and regional and reconstructive surgery include intraoral soft tissue changes, loss of proprioceptive sensitivity, altered tongue mobility, changes in the masticatory muscles and irregularities in bone contour. If, in addition, radiotherapy is applied, the patients develop xerostomia and mucosal atrophy, and most patients are unable to wear removable prostheses. Dental restoration is not only beneficial for chewing and nutrition but also favors speech and facial aesthetics. The achievement of optimal function in oncologic patients with SCCA of the tongue and floor of the mouth requires good lingual mobility, adequate facial competence, the cheek suction effect and dental rehabilitation with osseointegrated implants [6]. The purpose of this study was to evaluate the functional outcomes of oncologic patients diagnosed with intraoral SCCA who underwent resection of the tongue and floor of mouth and immediate reconstruction with a FAMM flap and osseointegrated implants. The use of the FAMM flap is the ideal reconstructive technique for the reconstruction of medium-sized defects involving the floor of the mouth and up to one third of the ipsilateral tongue. Although it provides a limited width and requires a second-stage procedure in many patients, it offers many benefits: (1) it is a thin and pliable flap with a great arc of rotation and length; (2) it is a musculo-mucosal flap ideal for reconstruction of mucosal defects with the absence of hair; (3) it is a safe and reliable flap that withstands postoperative radiotherapy [1]; (4) it is easy to harvest; (5) it enables primary closure of the donor site under 3 cm; (6) it can be harvested simultaneously with neck dissection; (7) previous neck dissection and radiation therapy are not contraindications [11,12,13]; (8) it can be superiorly or inferiorly based depending on the defect to be reconstructed; (9) it can be used as a reconstructive technique simultaneously with immediate placement of osseointegrated implants for both aesthetic and functional rehabilitation. Common regional flaps such as supraclavicular, submental and pectoralis major flaps are other reconstructive techniques suitable for these defects. Nevertheless, the pectoralis major flap provides excessive bulk and results in lingual fixation and aesthetic defects. The supraclavicular flap has an increased risk of partial flap necrosis, and the submental flap may compromise the neck dissection along with cancer ablation simultaneously [1,3]. In addition, these regional flaps may result in postoperative intraoral hair growth in male patients. On the other hand, the FAMM flap provides mucosal tissue for a like-to-like reconstruction and an optimal coating of the dental implants with mucosal tissue for long-term functional rehabilitation. 

In this study, the investigators demonstrate that for oncologic patients, areas affected by SCCA of the floor of the mouth and tongue can be aesthetically and functionally reconstructed with the FAMM flap and osseointegrated implants, allowing patients to achieve a life status similar to that prior to surgery. All patients developed medium-sized defects, and the defect sizes ranged from 3.7 × 2.1 cm to 6.3 × 4.2 cm, which meant they avoided microsurgical reconstruction. Despite the use of Duranceau’s technical modification [9], 68.2% of the patients required a two-stage procedure to take down and inset the flap pedicle, similar to previous studies [11,12]. Although the correct treatment of N0 neck is still debated, several articles support the elective neck dissection as the recommended treatment given the relevant rate of occult metastases [14,15,16]. All patients underwent selective neck dissection (levels 1–3 or 1–4) as part of their oncologic treatment, and the facial artery was preserved intact during neck dissection with no further complications. Complications such as wound dehiscence, hematoma and venous congestion were treated conservatively, and only one patient required surgical debridement due to distal flap necrosis. In this regard, O’Leary [12] and Massarelli [17] do not report a higher rate of flap complications with prior or concurrent neck dissections. Although radical neck dissections were not performed, the investigators believe that the viability of the FAMM flap simultaneously with a radical neck dissection should not be a problem as long as the facial artery is preserved. Although Ayad [11] reports good results for the harvest of inferiorly based FAMM flaps raised in patients with previously ligated facial arteries, the researchers recommend the preservation of the facial artery during neck dissection and the use of an alternative reconstructive option in patients for whom prior selective neck dissection has been performed or in patients for whom radical neck dissection is to be performed and the facial artery blood flow cannot be demonstrated. A limitation of this study is that previously irradiated patients have not been included. Although higher complication rates have been addressed in irradiated patients [11,12], future studies are needed to evaluate the outcome of FAMM flaps in these patients. 

Regarding the functional results of FAMM flap reconstruction, our study reveals a normal oral opening in 86.3% of patients, with excellent lingual tip elevation in 19 patients, excellent lingual protrusion in 15 patients and excellent lateral excursion in 14 patients. To achieve complete functional results, it is mandatory to rehabilitate the function of the speech articulation, deglutition and aesthetics with osseointegrated implants and implant-supported prostheses. To date, although the sample size is small, this is the first study that fully evaluates the functional outcomes of patients diagnosed with SCCA of the floor of the mouth and tongue reconstructed with a FAMM flap, osseointegrated implants and implant-supported prostheses. Complete functional rehabilitation requires dental rehabilitation with osseointegrated implants and implant-supported prostheses. Therefore, patients obtained not only improved aesthetics, but also speech pronunciation and chewing, allowing patients to recover a quality of life similar to that prior to surgery. An important topic to note would be the rehabilitation of speech articulation with a speech pathologist. Different studies have reported significant improvement in speech in different surgical specialties such as oral surgery and orthognathic surgery [7,8]. The interdisciplinarity of speech and its effectiveness in the treatment of oncologic patients require the cooperation of maxillofacial surgeons and speech pathologists to achieve the best possible result in terms of speech articulation. Our public health system allows the placement of osseointegrated implants in oncologic patients to achieve an overall treatment of the patients, and within the oncologic surgery section there is a multidisciplinary team for the oral rehabilitation of head and neck oncologic patients. Different studies have tried to analyze the functional results of patients reconstructed with a FAMM flap but none to date have included rehabilitated patients with osseointegrated implants. Benjamin [2] reports functional outcomes with intelligible speech, good tongue mobility, good swallowing and no mastication difficulties, but no dental rehabilitation was involved. Jowett [3] analyzes the functional results in terms of tongue mobility, oral competence, word articulation, upper lip and cheek sensations and the commissure position, but does not refer to dental rehabilitation. In our study, the 22 patients were rehabilitated comprehensively with osseointegrated implants and implant-supported prostheses. A total of 101 implants were placed, with a success rate of 92.1%, which allowed all patients to be rehabilitated. Of the 8 implants lost, 7 received radiotherapy, but this issue did not prevent the prosthetic rehabilitation of the patients. An important point of discussion would be the ideal time to place dental implants. Urken [18] and Navarro Cuéllar [19] demonstrate that there is a window period of 4–6 weeks between surgery and the beginning of radiotherapy, and another window period of 4–6 weeks between the beginning of radiotherapy and the appearance of an effect of radiotherapy on dental implants. As a consequence, by the time the effect of radiotherapy on the implants appears at 12 weeks, osseointegration of the implants would have already taken place. Therefore, we place the dental implants in the same surgical procedure of the ablative and reconstructive surgery. This leads to a faster prosthetic rehabilitation with better functional results, which allows patients to return to a quality of life similar to that prior to surgery. Future studies are needed to evaluate immediate or early loading of implants with implant-supported prostheses to allow functional rehabilitation for these patients immediately after surgery.

## 5. Conclusions

The use of the FAMM flap is an accurate and reliable reconstructive alternative for the reconstruction of small and medium-sized defects in oncologic patients with SCCA of the floor of the mouth and tongue. It is a predictable flap that allows a like-to-like reconstruction of the oral cavity with minimal morbidity, allowing simultaneous neck dissection to be performed with minimal complications. The immediate placement of osseointegrated implants and their further rehabilitation with implant-supported prostheses enables the functional rehabilitation of the patients, optimizing aesthetics, mastication and speech articulation even in patients undergoing irradiation, thus returning oncologic patients to a quality of life similar to that prior to surgery.

## Figures and Tables

**Table 1:** SCCA of tongue and floor of the mouth reconstructed with FAMM flap and dental implants.

G/A	Defect Size (cm)	TNM	Radiotherapy	N° Implants (Failure)	Complications	Mouth Opening	Lingual Mobility	Speech	Deglutition	Aesthetic Result
M/67	5.4 × 3.1	T2N1M0	Yes	4	Dehiscence donor site	N	E/E/E	N	2	E
M/59	5.2 × 3.3	T2N1M0	Yes	8 (2)	None	N	E/E/E	N	2	E
F/68	4.8 × 2.9	T2N0M0	No	4	None	N	E/G/G	N	2	G
M/59	5.5 × 3.4	T2N1M0	Yes	4	None	N	E/E/G	N	2	E
M/51	5.9 × 3.7	T2N2aM0	Yes	5(1)	Partial necrosis	PL	G/G/G	PI	1	G
F/64	3.8 × 2.7	T2N0M0	No	2	None	N	E/E/E	N	2	E
M/70	4.8 × 2.9	T2N0M0	No	5	None	N	E/E/E	N	2	E
M/59	5.7 × 3.6	T2N2bM0	Yes	12	Venous congestión	N	E/E/E	N	2	E
F/59	3.7 × 2.1	T2N0M0	No	2	None	N	E/E/G	N	2	E
M/63	5.3 × 3.2	T2N1M0	Yes	7(1)	None	PL	G/F/F	N	2	A
M/72	4.7 × 2.8	T2N1M0	Yes	5	None	N	E/E/E	N	2	E
F/59	5.2 × 3.7	T2N2aM0	Yes	2	None	N	E/E/E	N	2	E
M/64	5.8 × 3.4	T2N1M0	Yes	4	Venous congestión	N	E/E/E	N	2	E
M/68	4.3 × 3.2	T2N0M0	No	3	None	N	E/E/E	N	2	E
F/67	5.1 × 2.9	T2N0M0	No	4	None	N	E/E/E	N	2	G
M/51	4.7 × 3.1	T2N0M0	No	2	None	N	E/G/G	N	2	E
M/66	5.9 × 4.2	T2N2bM0	Yes	5(1)	Hematoma	N	E/E/E	N	2	E
F/64	3.9 × 3.1	T2N0M0	No	2	None	N	E/E/E	N	2	E
M/69	4.8 × 2.9	T2N0M0	No	4(1)	None	N	E/G/G	N	2	E
M/52	5.7 × 4.2	T2N2aM0	Yes	6(2)	None	N	E/G/F	N	1	E
F/59	6.3 × 4.2	T2N2bM0	Yes	5	Dehiscence donor site	PL	G/G/G	PI	1	E
M/63	5.7 × 3.9	T2N1M0	Yes	6	None	N	E/E/E	N	2	E

G/A: Gender/Age. TNM: T (tumor size), N (lymph nodes), M (metastasis).
